# Effects of Cycloplegic Agents on Ocular Parameters in Children with Myopia and Hyperopia

**DOI:** 10.1155/2023/9003942

**Published:** 2023-05-11

**Authors:** Guangzhi Qu, Bingfeng Wang, Saisai Ding, Xiaocui Liu, Lei Gao, Xuli Wang

**Affiliations:** ^1^Department of Ophthalmology, Weifang Eye Hospital, Zhengda Guangming Eye Group, Weifang, China; ^2^Zhengda Guangming International Eye Research Center, Qingdao University, Qingdao, China

## Abstract

**Background:**

To study the effect of cycloplegia on ocular parameters in children with myopia and hyperopia.

**Methods:**

Forty-two myopia and forty-four hyperopia eyes in children between 5 and 10 years of age were included. Measurements were taken before and after cycloplegia using 1% atropine sulfate ointment. The ocular parameters included central corneal thickness (CCT), corneal curvature (CC), anterior chamber depth (ACD), pupil diameter (PD), axial length (AL), and central retinal thickness (CRT).

**Results:**

There was no significant difference in CCT, CC, and CRT between the two groups without cycloplegia, but the ACD of the myopia (3.64 ± 0.28 mm) group was significantly higher than that of hyperopia (3.40 ± 0.24 mm; *t* = −4.522; *P* < 0.0001). The average PD of the myopia (4.85 ± 0.87 mm) group was significantly smaller than that of the hyperopia group (5.47 ± 1.15 mm; *t* = 2.903; *P* < 0.0046). The average AL of myopia (24.25 ± 0.77 mm) was significantly higher than that of hyperopia (21.73 ± 1.24 mm; *t* = 12.084; *P* < 0.0001). However, it was found that the average PD of myopia (7.68 ± 0.51 mm) was significantly larger than that of hyperopia (7.41 ± 0.57 mm; *t* = 2.364; *P*=0.0202) under cycloplegia. As for the changes in refractive factors before and after cycloplegia, deepened ACD and enlarged PD were noted in both the groups after cycloplegia.

**Conclusions:**

Cycloplegia not only affects ACD and PD but also leads to the reversal of PD differences between the two groups. Cycloplegia effects enabled us to study changes in all known ocular parameters in a short period.

## 1. Introduction

Myopia has not only become a public health problem of increasing concern in the world but also a “national disease” in China. The rapidly rising myopia prevalence, which has reached epidemic levels among middle and high schoolers in recent decades, has triggered renewed concerns about protecting Chinese students' vision. On 30 August 2018, eight departments of the Chinese government, including the Ministry of Education and National Health Commission, jointly resolved to implement the comprehensive plan to prevent nearsightedness among children and teenagers [1].

Myopia has plagued human beings for more than three centuries, but its specific pathogenesis has not been elucidated. Excessive near work is one of the most commonly known environmental factors relating to myopia in terms of perception. However, the precise mechanism of structural changes of the myopia eye is not well understood. Indeed, a significant transient elongation of axial length during short periods of accommodation has been observed in young adults [2–5]. Similar findings were found in our previous study about 20 years ago [6]. This study aimed to explore the effect of cycloplegia on ocular parameters for myopic and hyperopic children, and the analysis was performed to test differences between the two refractive groups.

## 2. Methods

### 2.1. Subjects

This study was approved by the Institutional Review Board of Weifang Eye Hospital and was conducted in accordance with the Declaration of Helsinki. Informed consent was obtained from their parents. Considering that the children enrolled were too young and have a state of hyperopia, the cycloplegia agent used in this study was 1% atropine sulfate ointment (Shenyang Xingqi Eye Medicine Co, Ltd.) twice a day for 5 days at home. All the ocular parameter measurement was taken at least 12 hours after the last administration of atropine ointment, in order to reduce the possible effect of the ointment on corneal curvature and thickness.

Children with myopia or hypermetropia after cycloplegia were included in the study, regardless of whether they had strabismus or astigmatism. All children in the study underwent a comprehensive ophthalmologic examination including slit-lamp biomicroscopy and fundoscopy. Exclusion criteria were active eye pathology, previous ophthalmic surgery, corneal opacities, glaucoma, retinal disease, and poor ocular fixation. No subjects had worn contact lenses ever. For children with binocular ametropia, the measurement data of the right eye are required to be included in the final analysis, and if ametropia is monocular, it is automatically included in the study.

### 2.2. Measurements

Children aged between five and ten years with myopia or hyperopia were recruited from the Optometry Clinic of Weifang Eye Hospital. The refractive state and corneal curvature were measured with the KR-1 auto kerato-refractometer (Topcon Corporation, Japan) after cycloplegia. Hypermetropia was defined as spherical equivalent refraction greater than 0.75 D. Myopia was defined as spherical equivalent refraction less than −0.50 D. Central corneal thickness before and after cycloplegia was obtained by Topcon Sp-3000p corneal endothelial cytometry (Topcon Corporation, Japan). Anterior chamber depth, pupil diameter, and axial length were measured by IOL master 500 (Carl Zeiss, Meditec, Germany). Central retinal thickness was automatically obtained with Heidelberg Spectralis OCT (Heidelberg Engineering, Germany). Three measurements were taken with each instrument under normal indoor lighting, and all the instruments are operated by experienced technicians and calibrated regularly to ensure their accuracy and repeatability.

### 2.3. Statistical Methods

All data were processed by using SPSS 20 software, and the chi-square test was used to compare the classified data. The measurement data were expressed as the mean ± standard deviation. Under the condition of normal distribution and homogeneity of variance, the independent and paired *T* tests were used. If the variance is not equal, the Mann–Whitney *U* test is used. A *P* value of <0.05 was considered statistically significant.

## 3. Results

A total of 86 eyes from 86 patients between the ages of 5 and 10 years were enrolled in this study, which included 42 myopia (19M, 23F) and 44 hyperopia patients (20M, 24F). The mean age of the myopia group was 8.4 ± 2.3 years, and the mean age of the hypermetropic group was 7.7 ± 2.1 years. There was no significant difference in the sex ratio (*χ*^2^ = 0.608; *P*=0.7378) and mean age (*t* = −1.540; *P*=0.1273) between the two groups. The mean diopter of the myopia group was −2.25 ± 1.36 D (95% CI −2.76 to −2.02 D) and that of the hyperopia group was 3.32 ± 2.94 D (95% CI 3.55 to 5.02 D).The results of a normality test showed that the distribution of all variables both before and after cycloplegia was normal (1 < skewness < 1).

### 3.1. Differences in Refractive Components between the Myopia Group and the Hyperopia Group before Cycloplegia

There were no significant differences in the mean central corneal thickness, corneal curvature, and central retinal thickness between the two groups. The mean anterior chamber depth of myopia was 3.64 ± 0.28 mm, which was significantly greater than that of hyperopia (3.40 ± 0.24 mm). The mean pupillary diameter of myopia (4.85 ± 0.87 mm) was significantly smaller than that of hyperopia (5.47 ± 1.15 mm). The mean axial length of myopic eyes (24.25 ± 0.77 mm) was significantly longer than that of hyperopic eyes (21.73 ± 1.24 mm, Table 1).

### 3.2. Differences in Refractive Components between the Myopia Group and the Hyperopia Group after Cycloplegia

The refractive components of the two groups were regained with the same measuring equipment after cycloplegia. The results showed that the differences in ocular parameters in the two groups except pupillary diameter were the same as those under noncycloplegia, but the pupillary diameter of myopia after cycloplegia (7.68 ± 0.51 mm) was significantly larger than that of hyperopia (7.41 ± 0.57 mm) after cycloplegia (Table 2).

### 3.3. Effects of Atropine on Refractive Components in the Myopia and Hyperopia Groups

Atropine significantly increased the anterior chamber depth and pupillary diameter in both the groups, while other refractive components had no significant changes (Table 3).

## 4. Discussion

Although the optical mechanism of myopia has long been understood, the research on the pathogenesis, natural course, and effective prevention of myopia has made slow progress. On the contrary, with the development of information society and the change in human eye habits, the increase in myopia is obvious and supported by epidemiology in terms of absolute and relative numbers [6–9]. The occurrence and development of myopia are closely related to intensity of reading and near work, which is almost the consensus of all ordinary people and clinical ophthalmologists. However, how reading and near work behavior induce the occurrence and development of myopia and the mechanism of pathological changes in myopia have not yet been clarified so far.

It is well known that, before presbyopia occurs in the eyes, our eyes can quickly switch between distant and near vision, the main known physiological mechanism is to initiate accommodation, and the amplitude of this accommodation decreases with age. The amplitude of accommodation decreased from about 15 D in early infancy to 1.00 D at the age of 60 [10]. Therefore, if we can obtain the difference in ocular parameters of the eye in far and near vision, it will help explore the impact of accommodation priming on ocular parameters of the eye. However, due to the limitations of today's measurement technology, it is not possible to obtain the instantaneous changes in all known ocular parameters in the two physiological states of far and near vision. To this end, we used the cycloplegic atropine ointment in this study, using it to simulate or even amplify the accommodation activity to study the impact of this “enlarged accommodation” on the known ocular parameters of the eye [6]. The data we obtained before cycloplegia represented the state of one eye in a near-viewing condition, whereas the data under cycloplegia represented that of one eye in a far-viewing condition. So it would be reasonable for us to learn the effect of “enlarged accommodation”—the amplitude of accommodation was enlarged by the application of atropine—on ocular parameters before and after cycloplegia. However, this “enlarged accommodation” would have an influence on ocular parameters similar to that of physiological or real accommodation from the viewpoint of lens changes, because the accommodation of human eyes mainly relies on the change of the lens in shape. This was fully proven in our experiment by the significant decrease in lens thickness after cycloplegia for each group [6]. As two diametrically opposed refractive states, we found that there was no significant difference in the central corneal thickness, corneal curvature, and central retinal thickness between myopia and hyperopia, except for the well-known difference in average AL. However, there were significant differences in the anterior chamber depth and pupil diameter.

### 4.1. Anterior Chamber Depth

Anterior chamber depth is undoubtedly an important ocular biometric parameter because the anterior chamber depth is located in the relative position of the lens. In this study, we found that the mean anterior chamber depth in myopia was significantly greater than that in hyperopia both before and after cycloplegia, and the anterior chamber depth was significantly deepened after cycloplegia in both the groups, which was consistent with our previous research conclusion of the anterior chamber depth measured by A-ultrasound [6]. Zhang et al. used IOL master to compare myopia and emmetropia in children of the same age. It was found that the anterior chamber depth in myopia was greater than that in emmetropia [11]. Considering that there is no significant difference in corneal curvature and central corneal thickness between the two groups in this study, we have reason to believe that the deeper anterior chamber depth in the myopia group may be closely related to the thinning of lens thickness [6]. Malyugin et al. studied the accommodative changes in the anterior chamber depth in patients with high myopia using anterior segment optical coherence tomography (AS-OCT). The results showed that accommodative changes in the ACD were significantly less pronounced in eyes with high myopia than those in emmetropic eyes, indicating that the change in lens configuration or thickness in myopia was less than that in emmetropia [12], which is also consistent with our previous study.

### 4.2. Pupil Diameter

The difference in PD between myopic and hyperopic eyes was significant (*t* = 2.903; *P* = 0.0046). Whether refractive error affects pupillary diameter was controversial [13–15]. Although Orr et al. suggested that refractive errors had no effect on the pupil diameter [15], studies with larger samples have confirmed that adult myopes have larger pupillary diameter than hyperopes [13, 14]. However, our study seems to be contrary to the above conclusions. In addition to age-group differences, our study did not receive the support based on children groups [16]. Guo et al. dynamically observed the pupil size of normal and myopia people with an infrared pupil tester and found that the pupil size of myopia was larger than that of emmetropia, and the pupil size increased with the deepening of myopia, especially in high myopia [17]. How to explain this seemingly contradictory phenomenon in the conclusion between our study and the above research? We note that the above three related studies all conducted under dark adaptation conditions, which is also supported by their average pupillary diameter obtained without cycloplegia. For example, the average pupillary diameter of children's myopia was 6.78 ± 0.81 mm [16] and that of adult myopia was 6.33 ± 0.82 mm [13] and 6.51 ± 0.8 mm [14]. The average pupil diameter of the three studies is closer to that of our myopia after cycloplegia but not 4.85 ± 0.87 mm before cycloplegia. It is well known that the size of the pupil is mainly determined by the intensity of light. It is regulated by the pupil sphincter and pupil dilator and through the sympathetic and parasympathetic nerves and the higher central *E*-*W* nucleus. Rosenfield et al. considered that myopic eyes have weak sympathetic or strong parasympathetic innervation, which may not only explain the smaller pupil size of myopic eyes than that of hyperopic eyes in natural conditions but also help understand the different effects of atropine [18].

### 4.3. Axial Length

Our previous studies on children's axial length were based on traditional A-ultrasound measurement, although the potential impact of corneal curvature was considered at that time, and the potential impact of the corneal curvature and central retinal thickness changes in the axial length were not considered. Therefore, in this study, we included all factors that potentially affect the measurement of the axial length, although no statistical validation was obtained to support our previous conclusions. However, the change in the average 30 um axial length of myopic eyes before and after cycloplegia and the corresponding statistical analysis (*t* = −1.976; *P*=0.0537) may reveal a similar change rule. Moreover, the effect of cycloplegia on the axial length has also been confirmed by some researchers. Raina et al. used Lenstar LS900 to study the axial length of 56 normal children aged 5 to 15 years and found that after 2% homatropine cycloplegia, the axial length of these children was significantly shortened compared with that before medication [19]. Unfortunately, the study did not focus on the refractive status of children. Tang X. P. and Tang Z. J. [20] used optical coherence biometry to measure the eye axis of children aged 4 to 11 years and found that the eye axis of the hyperopia group was significantly shortened after atropine cycloplegic treatment, while the eyes in the myopia group did not change significantly, and similar research conclusions were also supported by Wang et al. [21]. If the above studies were conducted using different cycloplegic agents, Woodman et al. used IOL Master to measure the effect of continuous 5 D accommodation near work for 30 minutes on the axial length of adult myopes and emmetropes and found that near accommodation could significantly increase their axial length [4]. Some people used LenStar to perform 3.00 and 4.50 D near stress tests on 35 adult emmetropic eyes and 37 myopic eyes and found that the axial lengths of the eyes in both the groups increased significantly [10].

In a word, from the point of view of the anatomical and physiological characteristics of human eyes, the only known refractive factor that can actively change instantaneously in visual activities is the change in the lens, which is caused by a series of mechanical changes involving intraocular muscles and has been confirmed by many studies. From the perspective of biological evolution, accommodation is the most primitive and basic visual control mechanism in the process of near reflex [22]. As long as we live in this three-dimensional world, retinal image defocus phenomenon is inevitable, and only eye accommodation can correct this defocus. Therefore, it may be a feasible way to study the changes in ocular parameters in a short time by studying the differences of refractive components in children and using the accommodative amplification effect of atropine.

## Figures and Tables

**Table 1:** Comparison of the mean values of refractive components before cycloplegia.

Refractive components	Myopia *x* ± *S*, CI	Hyperopia *x* ± *S*, CI	*T*	*P*
CCT (um)	521.74 ± 32.25	528.70 ± 34.85	0.955	0.3423
	511.69–531.79	517.97–539.42		

CC (D)	43.51 ± 1.40	43.54 ± 1.56	0.973	0.9227
	43.11–43.90	43.07–44.00		

ACD (mm)	3.64 ± 0.28	3.40 ± 0.24	−4.522	<0.0001
	3.56–3.72	3.32–3.47		

PD (mm)	4.85 ± 0.87	5.47 ± 1.15	2.903	0.0046
	4.60–5.11	5.12–5.81		

AL (mm)	24.25 ± 0.77	21.73 ± 1.24	−12.084	<0.0001
	24.03–24.46	21.36–22.10		

CRT (um)	217.23 ± 36.05	211.60 ± 18.95	−0.881	0.3809
	206.14–228.33	205.54–217.66		

CCT = central corneal thickness; CC = corneal curvature; ACD = anterior chamber depth; PD = pupillary diameter; AL = axial length; CRT = central retinal thickness; *x* ± *S* = mean ± standard deviation; CI = confidence interval.

**Table 2:** Comparison of the mean values of refractive components after cycloplegia.

Refractive components	Myopia *x* ± *S*, CI	Hyperopia *x* ± *S*, CI	*T*	*P*
CCT (um)	520.26 ± 32.56	527.82 ± 34.75	1.045	0.2988
	510.12–530.41	517.38–538.26		

CC (D)	43.01 ± 1.36	43.53 ± 1.56	1.726	0.0877
	42.63−43.40	43.07−44.00		

ACD (mm)	3.75 ± 0.24	3.63 ± 0.17	−2.356	0.0206
	3.68–3.82	3.59–3.69		

PD (mm)	7.68 ± 0.51	7.41 ± 0.57	−2.364	0.0202
	7.53–7.83	7.25–7.59		

AL (mm)	24.22 ± 0.76	21.73 ± 1.22	−12.137	<0.0001
	24.01−24.43	21.37−22.10		

CRT (um)	215.86 ± 28.84	213.51 ± 26.80	−0.381	0.7044
	206.99–227.74	204.83–222.20		

CCT = central corneal thickness; CC = corneal curvature; ACD = anterior chamber depth; PD = pupillary diameter; AL = axial length; CRT = central retinal thickness. *x* ± *S* = mean ± standard deviation; CI = confidence interval.

**Table 3:** Effects of atropine on refractive components in the myopia and hyperopia groups.

Refractive components	Before cycloplegia *x* ± *S*	After cycloplegia *x* ± *S*	*T*	*P*
M-CCT (um)	522.49 ± 32.28	520.68 ± 32.84	−1.332	0.1903
H-CCT (um)	528.70 ± 34.85	529.67 ± 34.44	0.924	0.3606
M-CC (D)	43.51 ± 1.40	43.01 ± 1.36	−1.809	0.0734
H-CC (D)	43.54 ± 1.56	43.53 ± 1.56	−0.364	0.7175
M-ACD (mm)	3.64 ± 0.28	3.75 ± 0.24	4.137	0.0001
H-ACD (mm)	3.40 ± 0.24	3.65 ± 0.16	13.057	<0.0001
M-PD (mm)	4.86 ± 0.88	7.68 ± 0.52	22.563	<0.0001
H-PD (mm)	5.47 ± 1.15	7.42 ± 0.57	11.535	<0.0001
M-AL (mm)	24.25 ± 0.77	24.22 ± 0.76	−1.976	0.0537
H-AL (mm)	21.73 ± 1.24	21.73 ± 1.22	0.339	0.7364
M-CRT (um)	217.23 ± 36.05	215.86 ± 28.84	−0.516	0.6086
H-CRT (um)	211.95 ± 19.07	213.51 ± 26.80	0.379	0.7066

*M* = myopic group; *H* = hyperopic group; CCT = central corneal thickness; CC = corneal curvature; ACD = anterior chamber depth; PD = pupillary diameter; AL = axial length; CRT = central retinal thickness; *x* ± *S* = mean ± standard deviation; CI = confidence interval.

## Data Availability

The data used to support the findings of this study are available from the corresponding author upon request.
